# Supplementary material for: Content-rich biological network constructed by mining PubMed abstracts
Source: BMC Bioinformatics. 2004 Oct 8;5:147. doi: 10.1186/1471-2105-5-147 (PMC528731; doi:10.1186/1471-2105-5-147)
Supplement: Additional File 5 — The original Chilibot query results of the term "long-term potentiation (LTP)" and 22 other terms, limiting the latest references analyzed to the years 1990, 1995, 2000, and 2004. [file 1471-2105-5-147-S5.bz2 › chilibotAdditionalFile5/ltp2000/html/ERK_NMDA.html]

 


 **ERK** and **NMDA** 
  
Found 16 abstracts in PubMed,  **16 abstracts were retrieved and analyzed**.  


---

 Search Google  |
 PDF files only 
|  EDU domain only 

---

**Interactive relationship** (e.g. stimulation, inhibition, etc)

- Activation of extracellular signal regulated kinase  **ERK**  has been shown to be necessary for  **NMDA**  receptor dependent long term potentiation LTP.  Ref: 10777769 J Neurosci, 2000
- Calcium influx via the  **NMDA**  receptor induces immediate early gene transcription by a MAP kinase  **ERK**  dependent mechanism.  Ref: 8757255 J Neurosci, 1996
- In cerebral cortical neurons, this calcium influx through  **NMDA**  receptors activates Ras and its downstream effector,  **Erk** , via nitric oxide NO generation by calcium dependent neuronal NO synthase.  Ref: 10588362 Diabetes Res Clin Pract, 1999
- Glutamate receptors of  **NMDA**  subtypes also participated in  **ERK**  activation, as shown after injection of the  **NMDA**  receptor antagonist MK 801.  Ref: 11102476 J Neurosci, 2000
- However, SAPK and  **ERK**  can also be coordinately activated in neurons in response to glutamate stimulation of  **NMDA**  receptors.  Ref: 10349832 J Neurochem, 1999
- **NMDA**  receptor mediated signaling is more responsible for the activation of the MKK6 p38 pathway than the MEK1  **ERK**  pathway.  Ref: 11108991 Neurosci Lett, 2000
- Increasing extracellular calcium enhanced  **ERK**  activation but reversed SAPK activation, further distinguishing the calcium dependencies of these two  **NMDA**  receptor mediated effects.  Ref: 10349832 J Neurochem, 1999
- Overall, our observations indicate that activation of the  **ERK**  signaling cascade is necessary for  **NMDA**  receptor independent LTP in area CA1 but not in area CA3 and suggest a divergence in the signaling cascades underlying  **NMDA**  receptor independent LTP in these hippocampal subregions.  Ref: 10777769 J Neurosci, 2000
- In addition, the activation of pip92 by  **NMDA**  was mediated by activation of mitogen activated protein kinases MAPKs, such as c Jun N terminal kinase JNK and p38 kinase, but not extracellular signal regulated kinase  **ERK**  in the mouse hippocampus and immortalized rat hippocampal progenitor cells.  Ref: 10854241 J Neurochem, 2000
- These results suggests that  **NMDA**  receptors can bidirectionally control  **ERK**  signaling.  Ref: 11062237 J Biol Chem, 2001
- Pretreatment with EGTA to eliminate extracellular calcium, or with APV, an  **NMDA**  receptor antagonist, to inhibit calcium influx through the  **NMDA**  receptor, attenuated the activation of  **ERK** .  Ref: 10673387 Biochem Biophys Res Commun, 2000
- The chronic morphine induced increases in  **ERK**  activity and TH expression in the VTA also were blocked by local infusion of  **NMDA**  glutamate receptor antagonists, suggesting a role for glutamate in mediating these drug effects.  Ref: 8764658 J Neurosci, 1996

**Parallel relationship** (e.g. studied together, co-existance, homology, etc.)

- The mechanism by which changes in cytoplasmic calcium mediate  **NMDA**  induced activation of Ras and  **Erk**  is not known.  Ref: 10588362 Diabetes Res Clin Pract, 1999
- N Methyl D aspartate  **NMDA**  receptor activation of extracellular signal regulated kinase  **ERK**  was examined in primary cortical cultures.  Ref: 11062237 J Biol Chem, 2001
- AMPA... caused an association of a G protein beta subunit with a Ras, Raf kinase, and MAP  **ERK**  kinase MEK 1 complex.  Ref: 7559406 J Biol Chem, 1995
- These latter bring about on the one hand a raising of calcium i partly due to the potentiation of the  **NMDA**  receptor response to endogenous glutamate and on the other hand the activation of certain MAP kinases  **ERK**  and JNK which lead to the initiation of the cell death program.  Ref: 9312350 Bull Acad Natl Med, 1997
- In primary cultures of striatal neurons, glutamatergic activation of  **ERK**  and one of its transcription factor targets, CREB, showed a calcium dependence typical of  **NMDA**  receptor mediated responses.  Ref: 10349832 J Neurochem, 1999
- We studied the role of  **ERK**  in three forms of  **NMDA**  receptor independent LTP LTP induced by very high frequency stimulation 200 Hz LTP, LTP induced by the K channel blocker tetraethylammonium TEA TEA LTP, and mossy fiber MF LTP MF LTP.  Ref: 10777769 J Neurosci, 2000
